# Survivin expression and serum levels in pancreatic cancer

**DOI:** 10.1186/s12957-015-0605-7

**Published:** 2015-05-28

**Authors:** He Dong, Dongmeng Qian, Yaqiu Wang, Lingsheng Meng, Dong Chen, Xiangyu Ji, Wei Feng

**Affiliations:** Department of Anesthesia, The affiliated hospital of Qingdao University, Qingdao, China; Department of Microbiology, Qingdao University, Qingdao, Shandong China; Department of Clinical Laboratory, Qingdao Women and Children’s Hospital, Qingdao, China; Department of Clinical Laboratory, People’s Hospital of Zhangqiu, Jinan, China

**Keywords:** Pancreatic ductal adenocarcinoma, Prognosis, Survivin

## Abstract

**Background:**

Survivin, an inhibitor of apoptosis, is overexpressed in pancreatic ductal adenocarcinoma (PDAC). Its expression is known to be associated with poor clinical outcome. However, to our knowledge, there has been no study to characterize its usefulness as a serum marker for human pancreatic cancer. Furthermore, the relation between survivin expression and the serum level of survivin has not been widely studied in PDAC. We performed this study to investigate the expression and serum level of survivin in PDAC and its clinical significance as a prognostic factor.

**Methods:**

We performed immunohistochemical staining for survivin in formalin-fixed, paraffin-embedded blocks from 80 PDAC tissues. The serum level of survivin from the patients (*n* = 80) and age-matched healthy volunteers (*n* = 80) were analyzed by enzyme-linked immunosorbent assays (ELISAs) prior to surgical resection. Levels of expression were correlated with clinicopathological parameters.

**Results:**

Serum survivin concentrations were significantly elevated in patients with PDAC when compared with healthy sera (*P* < 0.001). High serum survivin levels were significantly associated with perineural invasion, venous invasion, lymph node status (N stage), cell differentiation, and recurrence but not with the tumor size, age, gender of the patients, or tumor location. The median overall survival time of the group with normal serum survivin levels was longer than that of the group with elevated serum survivin. The independent factors associated with overall survival were advanced pancreatic cancer and elevated serum survivin level. Of the 80 cases of PDAC, 65 (81.25 %) were positive for survivin expression. There were significant associations between survivin expression and perineural invasion, venous invasion, and lymph node status. A significant difference in overall survival was associated with survivin expression.

**Conclusions:**

Patients with elevated serum survivin level and high survivin expression at diagnosis demonstrated a poor outcome. Detection of serum survivin or tissue survivin expression may predict the prognosis of patients with PDAC.

## Background

Pancreatic ductal adenocarcinoma (PDAC) is the fourth leading cause of cancer-related death in Western countries and has the poorest survival rate (<5 %) among the common malignancies [1, 2]. Although surgical resection shows promise as an effective treatment for PDAC, a lack of effective tools for diagnosis in the earliest stages results in low 5-year survival rates, which drop rapidly from >50 % in patients with stage I to <5 % in patients with more advanced stages of the disease [3–5]. Both imaging techniques and serological markers, such as carbohydrate antigen 19-9 (CA19-9) and carcinoembryonic antigen (CEA), have used for many years for diagnosis of PDAC, however, it is still difficult to distinguish patients with chronic pancreatitis (CP), a high-risk population for PDAC, from those with PDAC [6–9]. Therefore, to improve the prognosis of PDAC, the development of specific and noninvasive biomarkers for PDAC diagnosis is required and especially for early-stage tumors.

Survivin is a member of the inhibitor of apoptosis protein (IAP) family originally discovered in the baculovirus [10]. Structurally, survivin is a unique IAP protein [11], organized as a stable dimer [12] containing only one baculoviral IAP repeat domain and a –COOH terminus coiled-coil domain [13]. Survivin is unique for its expression in a wide range of embryonic and fetal tissues but is undetectable in terminally differentiated normal adult tissues. However, it is re-expressed in transformed cell lines and several human cancer cells at a frequency of 34–100 % [13, 14]. As a prognostic factor, survivin expression is significantly associated with poor clinical outcome in cancers such as neuroblastoma, colorectal cancer, breast cancer, lung cancer, esophageal cancer, medulloblastoma, and pediatric acute lymphoblastic leukemia [15–22].

Survivin expression in pancreatic cancer has been widely studied. Satoh et al. reported that expression of survivin may be upregulated during an early stage of tumorigenesis, and it may be involved in the development of cancer by reducing cancer cell apoptosis [23]. Kami and Ekeblad et al. reported that expression of survivin in pancreatic cancer tissues could be a useful prognostic marker in patients with this cancer [24, 25]. Lee et al. found that survivin expression seems to have potential as a predictive marker for the response to chemotherapy [26].

Although elevated expression of survivin has been found in many cancer tissues, including PDAC tissues, and its association with tumor behavior and patient prognosis has been reported, few studies have analyzed the serum levels of survivin in patients with PDAC. Furthermore, the relationship between survivin expression and serum survivin level has not been widely studied in PDAC.

In the present work, we determined the serum levels of survivin in patients with PDAC, then performed immunohistochemical staining for survivin in PDAC tissues. Survivin expression and serum survivin levels were correlated with clinicopathological parameters.

## Methods

### Patients and clinical data

For this study, blood sera (*n* = 80) of patients with PDAC and the sera of 80 controls were collected from January 1, 2007 to December 30, 2012. All blood samples were collected before any therapeutic procedures, including surgery, chemotherapy, and radiotherapy. Blood samples were taken directly before surgery. After centrifugation of the peripheral blood, serum samples were stored at −20 °C until assayed. A diagnosis of PDAC was confirmed by histological examination or fine-needle aspiration cytology. Histological typing of the tumors was performed according to World Health Organization (WHO) criteria. All the enrolled patients with PDAC showed the tumor histotype of pancreatic ductal adenocarcinoma. The tumors were staged according to the sixth edition of the American Joint Committee on Cancer tumor–node–metastasis (TNM) system. Overall survival time was calculated from the date of operation to the date of death or last follow-up. Patients who did not survive the first 30 days after surgery were excluded from the survival analysis. The patients’ clinicopathological characteristics are summarized in Table 1. Their median age was 46 years (range, 32–71 years); 54 patients had stage I and II disease, 18 patients had stage III disease, and 8 had stage IV disease. Eighteen patients underwent only palliative resection and were treated with combination chemotherapy comprising epirubicin, cisplatin, and 5-fluorouracil (5-FU). Of 47 patients who had curative surgery, 25 underwent adjuvant treatment with radiotherapy. Recurrent cancer developed in 28 patients, and the median time to recurrence was 163 days (range, 51–1370 days).

**Table 1:** Patients’ clinicopathological characteristics

Clinicopathologic characteristics	*n* = 80
Gender	
Male	62
Female	18
Age (years)	
Median	46
Range	32–71
Tumor location	
Head	55
Body	3
Tail	7
Body and tail	15
Tumor differentiation	
Well	10
Moderate	57
Poor	8
Others	5
Stage	
I	17
II	37
III	18
IV	8
Lymphatic invasion	
Positive	47
Negative	33
Perineural invasion	
Yes	55
No	25
Venous invasion	
Yes	50
No	30
Recurrence	
Yes	56
No	24

The 80 healthy controls were recruited at the Healthy Physical Examination Center of the affiliated hospital of the medical college of Qingdao University. The health condition checkup included a detailed history; physical, radiological, and endoscopic examinations; blood tests; tumor marker tests (CA19-9, CEA); and abdominal sonography. Participants with no evidence of pancreatic disease or other abnormalities were enrolled as cancer-free controls.

The study was approved by the Ethics Committee of the affiliated hospital of the medical college of Qingdao University. Written consent for using the samples for research purposes was obtained from all patients and controls prior to surgery or blood sample collection.

### Immunohistochemical staining

Formalin-fixed, paraffin-embedded blocks from the 80 patients with surgically resected PDAC tissues and 24 samples of normal pancreas tissue were studied. Consecutive tissue sections were stained for survivin. Known positive and negative control slides were used to determine the acceptability of the staining reaction. For the stains, cytoplasmic immunoreactivity was considered a positive reaction. Samples with <10 % positive cells were recorded as showing negative expression and those with ≥10 % positive cells were recorded as showing positive expression. All samples were reviewed independently by two pathologists.

### Measurement of serum survivin levels

Survivin (Quantikine Human survivin Immunoassay, R&D System, USA) concentrations were determined by using an enzyme-linked immunosorbent assay (ELISA) method according to the manufacturer’s instructions. All specimens were assayed twice .The minimum detectable dose (MDD) of survivin ranged from 1.58 to 9.96 pg/ml.

### Statistical analysis

All statistical analyses were performed using SPSS statistical software (Chicago, IL, USA). Data are presented as means (SE). We used Mann–Whitney U-test to compare differences in the serum survivin concentrations between the cancer patients and the control group. The relationship between serum and tissue survivin level was assessed by Fisher exact test. The relationship between serum or tissue survivin level with the clinicopathological parameters was assessed by χ^2^ analysis. For continuous variables not normally distributed, the variables are given as median (25th–75th percentile) and they were compared using the Mann–Whitney U-test. Survival curves were plotted using the Kaplan–Meier method. The difference in survival between groups was compared using the log-rank test.

The prognostic factors were examined by univariate and multivariate analyses (Cox proportional hazards regression model). *P* < 0.05 was considered to indicate statistical significance.

## Results

### Serum levels of survivin

**Fig. 1:**
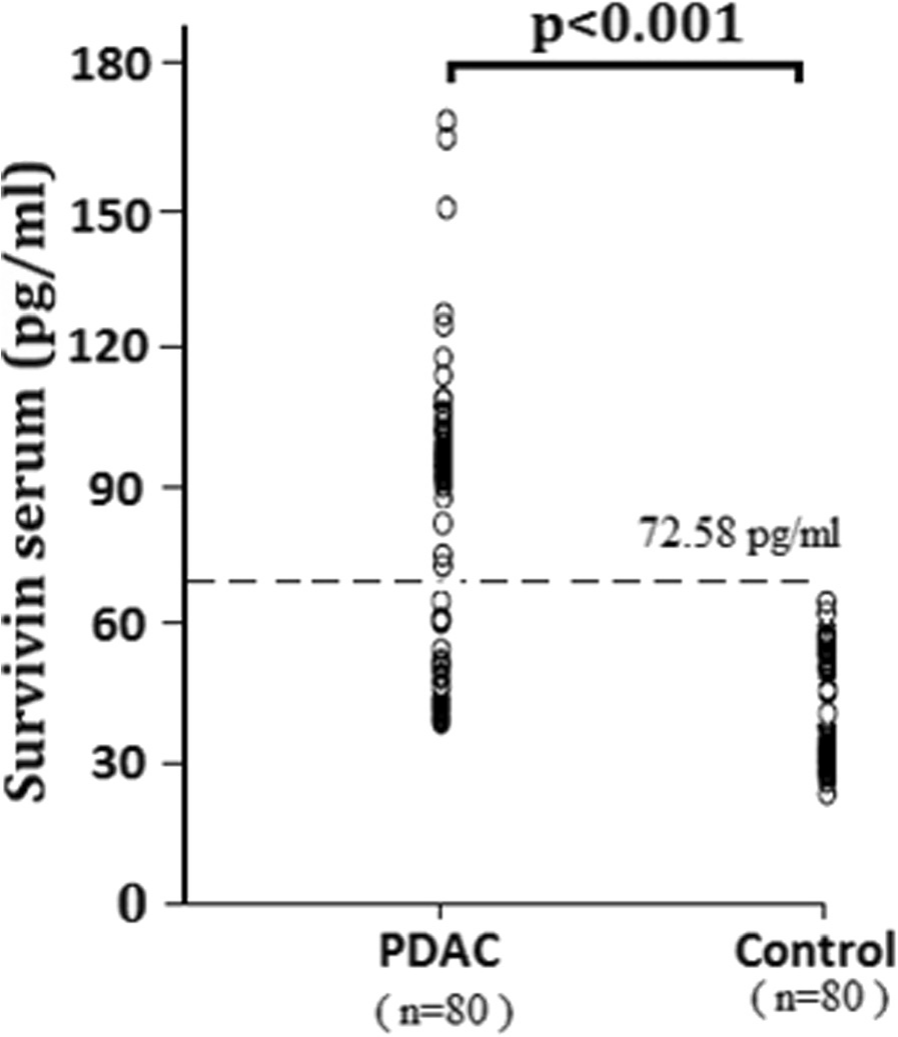
Comparison of serum survivin levels in healthy controls and patients with PDAC. The *horizontal line* in each plot represents the cutoff value. ^*^ *P* < 0.001

In the healthy controls, the median serum level of survivin were 41.36 (19.4–72.58) pg/ml, which was significantly lower than that in patients with PDAC 99.4 (46.4–176) pg/ml (Fig. 1, *P* < 0.001).The cutoff value was set at 72.58 pg/ml (75th percentile of the survivin distribution in the controls). Higher serum survivin levels (≥72.58 pg/ml) were found in 63.75 % (51/80) of patients with PDAC, and 36.25 % of patients (29/80) had levels lower than 72.58 pg/ml.

### Expression of survivin in PDAC

**Fig. 2:**
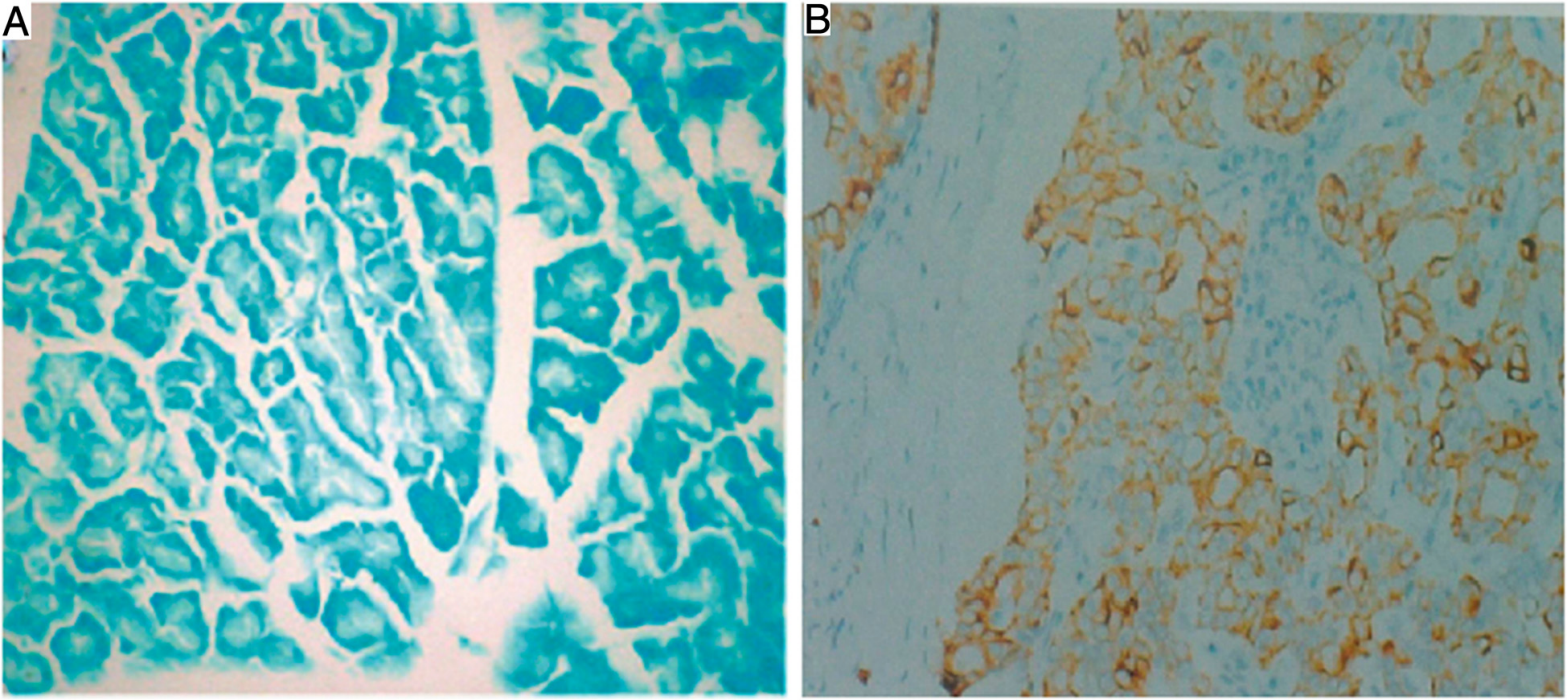
Expression of survivin in normal pancreas **a** and PDAC **b** (×200)

Eighty PDAC specimens and 24 normal pancreatic samples were screened for survivin protein expression. Overexpression of survivin was found in 65 cases (81.25 %) among the 80 PDAC tissue specimens, in which immunostaining was predominantly seen in the cytoplasm of tumor cells (Fig. 2). No positive staining was found in the 24 samples of normal pancreas, which was significantly different from the level in patients with PDAC (*P* < 0.001).

### Relation between tissue survivin and serum survivin

Among the 65 (81.25 %) of the 80 patients with PDAC whose tissues showed positive survivin expression, increased serum survivin levels (≥72.58 pg/ml) were found in 61.5 % (40/65). Among the 15 (8.75 %) of the 80 patients with PDAC whose tissues were negative for survivin expression, increased serum survivin levels (≥72.58 pg/ml) were found in 13.3 % (2/15). A significant association was found between serum survivin level and survivin expression (Table 2, *P* < 0.01).

**Table 2:** Relation between tissue and serum survivin

	Serum survivin		
Survivin expression	≥72.58 pg/ml (*n* = 51)	<72.58 pg/ml (*n* = 29)	*P*-value
Positive (*n* = 65)	40	25	<0.01
Negative (*n* = 15)	2	13	

### Serum and tissue survivin levels and clinicopathological features in patients with PDAC

Using 72.58 pg/ml as the cutoff value, the patients with PDAC were divided into group A (*n* = 51), comprising those with the higher serum level of surviving (≥72.58 pg/ml) and group B (*n* = 29) comprising those with a lower level (<72.58 pg/ml). The χ2 analysis showed that the preoperative serum survivin levels correlated well with perineural invasion, venous invasion, lymph node status (N stage), histologic grade, and tumor stage but not with the tumor size, age, gender, and lymph node status (N stage) of the patients or tumor location (Table 3). Spearman rank correlation analysis revealed a correlation between serum survivin level and tumor stage, perineural invasion, venous invasion, recurrence, and histological differentiation (Table 4). These observations support the hypothesis that the progression of PDAC is associated with increased serum survivin levels.

**Table 3:** Relationship between serum levels or tissue survivin expression and clinicopathological factors

	Serum survivin			Survivin expression		
Group	≥72.58 pg/ml	<72.58 pg/ml	*P*-value	Positive	Negative	*P*-value
Age(years)			NS			NS
<50(*n* = 32)	23(71.8 %)	9(28.2 %)		20(62.5 %)	12(37.5 %)	
>50(*n* = 48)	28(58.3 %)	20(41.7 %)		31(64.6 %)	17(35.4 %)	
Gender			NS			NS
M(*n* = 62)	40(64.5 %)	22(35.5 %)		37(59.7 %)	25(40.3 %)	
F(*n* = 18)	11(61.1 %)	7(38.9 %)		14(77.8 %)	4(22.2 %)	
Location			NS			NS
Head(*n* = 55)	34(61.8 %)	21(38.2 %)		32(58.2 %)	23(41.8 %)	
Body(*n* = 3)	3(0 %)	0(100 %)		2(66.7 %)	1(33.3 %)	
Tail(*n* = 7)	4(57 %)	3(43 %)		5(71.4 %)	2(28.6 %)	
Body and tail(*n* = 15)	10(66.7 %)	5(33.3 %)		12(80 %)	3(20 %)	
Stage(TNM)			<0.01			NS
I(*n* = 17)	7(41.2 %)	10(58.8 %)		10(58.8 %)	7(41.2 %)	
II(*n* = 37)	23(62.1 %)	17(37.9 %)		24(64.9 %)	13(35.1 %)	
III(*n* = 18)	15(83.3 %)	3(16.7 %)		17(94.4 %)	1(5.5 %)	
IV(*n* = 8)	8(100 %)	0(0 %)		5(62.5 %)	3(37.5 %)	
Lymphatic invasion			<0.01			<0.01
Positive(*n* = 47)	39(83 %)	8(17 %)		36(76.6 %)	11(23.4 %)	
Negative(*n* = 33)	12(36.3 %)	21(63.7 %)		25(75.7 %)	8(24.3 %)	
Perineural invasion			<0.01			<0.01
Yes(*n* = 55)	44(80 %)	11(20 %)		39(70.9 %)	16(29.1 %)	
No(*n* = 25)	7(28 %)	18(72 %)		12(48 %)	13(52 %)	
Venous invasion			<0.01			
Yes(*n* = 50)	40(80 %)	10(20 %)		37(74 %)	13(26 %)	<0.01
No(*n* = 30)	11(36.6 %)	19(63.4 %)		14(46.7 %)	16(53.3 %)	
Cell differentiation			<0.01			NS
Well(*n* = 10)	2(20 %)	8(80 %)		4(40 %)	6(60 %)	
Moderate(*n* = 57)	37(64.9 %)	20(35.1 %)		42(73.7 %)	15(26.3 %)	
Poor(*n* = 8)	8(100 %)	0(0 %)		4(50 %)	4(50 %)	
Others(*n* = 5)	4(80 %)	1(20 %)		1(20 %)	4(80 %)	
Recurrence			<0.01			NS
Yes(*n* = 56)	45(80.3 %)	11(19.7 %)		33(58.9 %)	23(41.1 %)	
No(*n* = 24)	6(25 %)	18(75 %)		18(75 %)	6(25 %)	

*P* determined by χ^2^ test. *NS* no significance

**Table 4:** Spearman correlation analysis between serum survivin levels and clinicopathological factors

Variable	Serum survivin level		Survivin expression	
	Spearman correlation	*P*-value	Spearman correlation	*P*-value
Age	–0.09	NS	−0.076	NS
Gender	–0.12	NS	−0.17	NS
Location	0.07	NS	−0.08	NS
Stage (TNM)	0.783	<0.0001	0.854	<0.001
Lymphatic invasion	0.656	<0.01	0.527	<0.01
Perineural invasion	0.430	<0.001	0.380	<0.01
Venous invasion	0.586	<0.0001	0.783	<0.01
Cell differentiation	0.52	<0.0001	0.14	NS
Recurrence	0.12	NS	0.07	NS

Of the samples examined for survivin immunohistochemical staining, survivin was significantly associated with lymphatic invasion, perineural invasion, and venous invasion (Table 3). Spearman analysis revealed a correlation between positive survivin expression and TNM stage, perineural invasion, and venous invasion (Table 4). These observations support the hypothesis that the progression of PDAC is associated with positive survivin expression.

### Association between serum level and tissue expression of survivin and prognosis and survival rates for patients with PDAC

**Fig. 3:**
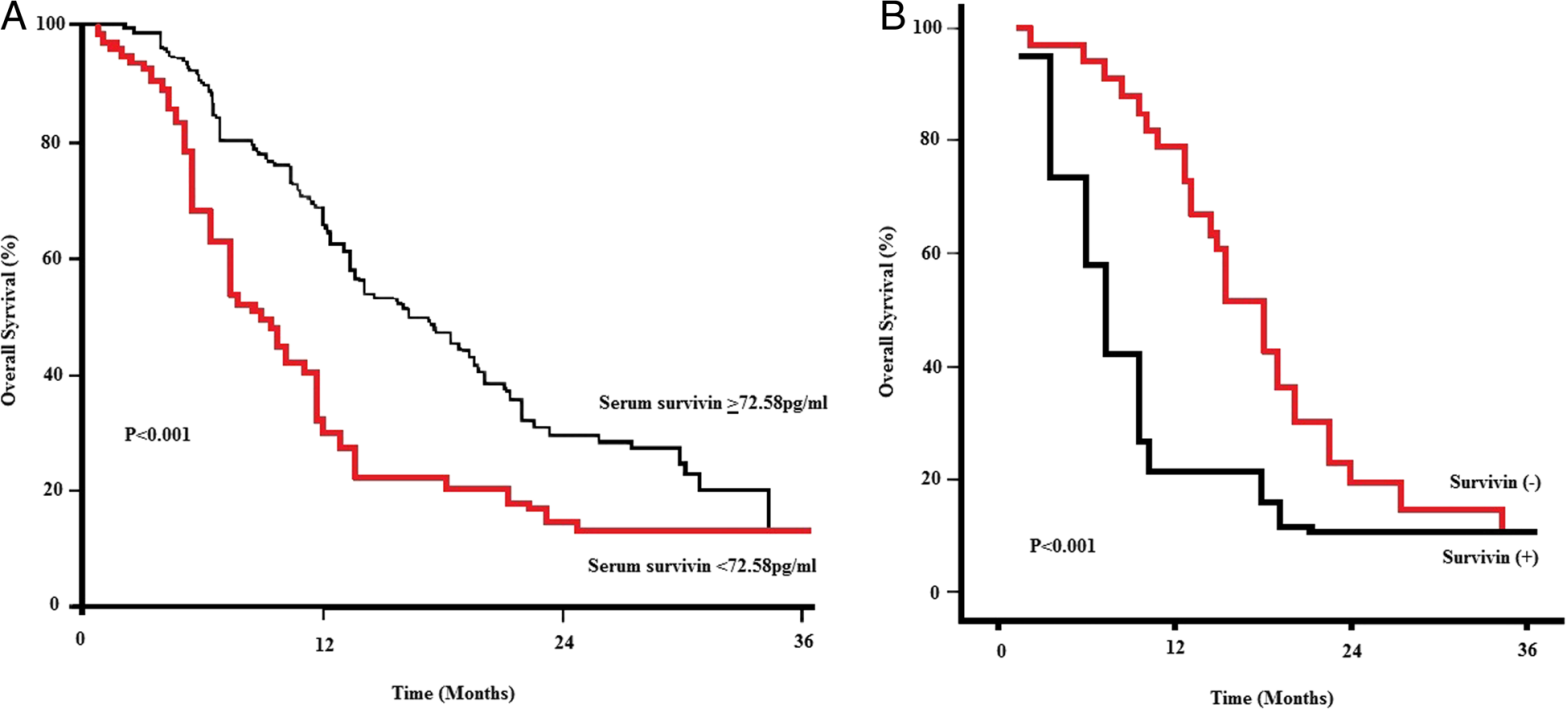
Serum **a** and tissue survivin **b** corresponded with the progression of PDAC

A log-rank test and Kaplan–Meier analysis were used to calculate the effect of serum levels and tissue expression of survivin on survival. The log-rank test showed that serum and tissue survivin correlated strongly with the patients’ survival time (*P* < 0.001; Fig. 3a, b).

More specifically, the median survival time of patients with high serum levels of survivin was only 9 months (positive tissue survivin was 8.8 months), whereas the median survival time of those with low levels of survivin was 26 months (negative tissue survivin was 26.3 months). The cumulative 5-year survival rate was 38.4 % in the group with low serum levels of survivin (*n* = 29) and 37.6 % in the group whose tissues were negative for survivin. In the group with high serum levels of survivin (Fig. 3a), the 5-year survival rate was only 9.28 %, and it was 9.63 % in the group whose tissues were positive for survivin (Fig. 3b).

Clinical stage, serum and tissue levels of survivin, tumor classification, histological differentiation, perineural invasion, and venous invasion were analyzed using univariable and multivariable Cox regression analyses. Univariable analyses revealed that clinical stage, serum and tissue levels of survivin, and histological differentiation were significant predictors of PDAC (Table 5). Multivariable analysis showed that clinical stage, serum and tissue levels of survivin, and histological differentiation were independent predictors for PDAC on the basis of changes in likelihood interactions between the parameters listed in univariable regression analyses (Table 5).

**Table 5:** Univariate and multivariate analysis of various prognostic variables in patients with PDAC

	Univariate analysis		Multivariate analysis	
Characteristic	Regression coefficient(SE)	*P*-value	HR (95 % CI)	*P*-value
Serum survivin	0.317(0.042)	<0.001	2.89(1.98–5.83)	0.003
Survivin expression	0.302(0.018)	<0.001	2.65(1.74–5.25)	0.006
Stage (TNM)	0.156(0.06)	0.027	2.74(1.56–5.14)	0.014
Lymphatic invasion	0.178(0.013)	0.042	1.54(1.12–4.34)	0.015
Perineural invasion	0.65(0.18)	0.012	0.84(0.53–1.27)	0.002
Venous invasion	0.54(0.12)	0.034	2.34(1.48–5.13)	0.021
Cell differentiation	0.74(0.28)	<0.01	2.46(1.75–5.54)	0.004
Recurrence	0.33(0.18)	0.093	1.64(1.15–3.38)	0.084

*HR* hazard ratio, *CI* confidence interval

## Discussion

Identification of targets for early detection of PDAC is important to improve the prognosis of patients with this pernicious disease. Currently, carcinoembryonic antigen and cytokeratin-19 fragments are routinely used as serum markers for detection of PDAC. Owing to the low sensitivity and specificity of detection of these markers, additional serum markers must be established for early detection and diagnosis of PDAC.

Survivin, an IAP, has been studied as a prognostic marker in various cancers. Adida et al. reported that survivin expression in neuroblastoma correlated with unfavorable histology and aggressive and disseminated disease [15]. In colorectal cancer and breast cancer, patients with surviving-positive tumors had a decreased apoptotic index and worse survival rates than those with survivin-negative tumors [17, 27]. In addition, survivin expression was correlated with poor prognosis in esophageal cancer and non-small cell lung cancer [19, 20]. Survivin expression in pancreatic cancer tissues could also be a useful prognostic marker in patients with this disease [24, 25].

In the present study, we showed that survivin levels in serum samples from patients with PDAC are significantly higher than those in a control population of healthy blood donors. Notably, serum survivin levels were higher in patients with perineural invasion, poor lymph node status, and venous invasion, compared with those without. We also found that positive survivin expression was higher in patients with PDAC with perineural invasion, poor lymph node status, and venous invasion, compared with those without. Thus, survivin concentrations and tissue survivin expression may be suitable as routine serum and tissue markers for the detection of metastatic disease in PDAC.

In addition, the serum survivin level was much higher in patients with PDAC at advanced stages and those with recurrence and poor differentiation; no significant differences in the serum levels of survivin were found in PDAC patients with different ages, genders, and tumor sizes. Thus, measurement of the serum level of survivin is useful for predicting the prognosis of patients with PDAC. However, no relation was found between survivin expression, TNM stage, recurrence, and tumor differentiation. This may have been caused by a small sample size.

Evaluation of the survival data showed that survival time was significantly shorter in patients with positive survivin expression and survivin concentrations >72.58 pg/ml. Thus, high survivin concentrations and positive survivin expression predicted a poor prognosis for patients with PDAC, especially in those presenting with metastasis. In addition, the uni- and multivariate analyses in the present study showed the prognostic significance of clinicopathologic factors such as TNM and tumor differentiation. These findings indicate that the results obtained from the present series of cases agree to PDAC in other counties. In addition, Cox regression analysis showed that survivin was an independent prognostic variable.

In the present study, we assessed the prognostic value of serum survivin in patients with PDAC and identified survivin as an independent prognostic factor. Owing to the limited number of controls and patients in this study, further studies are required. In addition, studies with serum samples and tissue survivin expression measured at different phases of PDAC progression could further elucidate the role of survivin in the growth and metastasis of PDAC.

## Conclusions

Our findings provide evidence that survivin has important roles at different phases of metastatic spread of PDAC, and that measurement of serum survivin or detection of survivin expression could be of clinical value when identifying patients at high risk for disease progression.

## References

[1] Jemal A, Siegel R, Ward E, Hao Y, Xu J, Murray T (2008). Cancer statistics, 2008. CA Cancer J Clin.

[2] Warshaw AL, Fernandez-del CC (1992). Pancreatic carcinoma. N Engl J Med.

[3] Li HY, Cui ZM, Chen J, Guo XZ, Li YY (2015). Pancreatic cancer: diagnosis and treatments. Tumour Biol.

[4] Surlin V, Bintintan V, Petrariu FD, Dobrin R, Lefter R, Ciobică A (2014). Prognostic factors in resectable pancreatic cancer. Rev Med Chir Soc Med Nat Iasi.

[5] Wagner M, Redaelli C, Lietz M, Seiler CA, Friess H, Buchler MW (2004). Curative resection is the single most important factor determining outcome in patients with pancreatic adenocarcinoma. Br J Surg.

[6] Lowenfels AB, Maisonneuve P, Cavallini G, Ammann RW, Lankisch PG, Andersen JR (1993). Pancreatitis and the risk of pancreatic cancer. International Pancreatitis Study Group. N Engl J Med.

[7] Talamini G, Falconi M, Bassi C, Sartori N, Salvia R, Caldiron E (1999). Incidence of cancer in the course of chronic pancreatitis. Am J Gastroenterol.

[8] Maisonneuve P, Lowenfels AB (2002). Chronic pancreatitis and pancreatic cancer. Dig Dis.

[9] Dzeletovic I, Harrison ME, Crowell MD, Pannala R, Nguyen CC, Wu Q (2014). Pancreatitis before pancreatic cancer: clinical features and influence on outcome. J Clin Gastroenterol.

[10] Deveraux QL, Reed JC (1999). IAP family proteins–suppressors of apoptosis. Genes Dev.

[11] Salvesen GS, Duckett CS (2002). IAP proteins: blocking the road to death’s door. Nat Rev Mol Cell Biol.

[12] Verdecia MA, Huang H, Dutil E, Kaiser DA, Hunter T, Noel JP (2000). Structure of the human anti-apoptotic protein survivin reveals a dimeric arrangement. Nat Struct Biol.

[13] Ambrosini G, Adida C, Altieri DC (1997). A novel anti-apoptosis gene, survivin, expressed in cancer and lymphoma. Nat Med.

[14] Cheung CH, Huang CC, Tsai FY, Lee JY, Cheng SM, Chang YC (2013). Survivin—biology and potential as a therapeutic target in oncology. Onco Targets Ther.

[15] Ito R, Asami S, Motohashi S, Ootsuka S, Yamaguchi Y, Chin M (2005). Significance of survivin mRNA expression in prognosis of neuroblastoma. Biol Pharm Bull.

[16] Islam A, Kageyama H, Takada N, Kawamoto T, Takayasu H, Isogai E (2000). High expression of Survivin, mapped to 17q25, is significantly associated with poor prognostic factors and promotes cell survival in human neuroblastoma. Oncogene.

[17] Goossens-Beumer IJ, Zeestraten EC, Benard A, Christen T, Reimers MS, Keijzer R (2014). Clinical prognostic value of combined analysis of Aldh1, survivin, and EpCAM expression in colorectal cancer. Br J Cancer.

[18] Xu C, Yamamoto-Ibusuki M, Yamamoto Y, Yamamoto S, Fujiwara S, Murakami K (2012). High survivin mRNA expression is a predictor of poor prognosis in breast cancer: a comparative study at the mRNA and protein level. Breast Cancer.

[19] Kato J, Kuwabara Y, Mitani M, Shinoda N, Sato A, Toyama T (2001). Expression of surviving in esophageal cancer: correlation with the prognosis and response to chemotherapy. Int J Cancer.

[20] Rosato A, Menin C, Boldrin D, Dalla Santa S, Bonaldi L, Scaini MC (2013). Survivin expression impacts prognostically on NSCLC but not SCLC. Lung Cancer.

[21] Abdel-Aziz A, Mohamed MA, Akl FM, Taha AN (2013). Survivin expression in medulloblastoma: a possible marker for survival. Pathol Oncol Res.

[22] Esh AM, Atfy M, Azizi NA, El Naggar MM, Khalil EE, Sherief L (2011). Prognostic significance of survivin in pediatric acute lymphoblastic leukemia. Indian J Hematol Blood Transfus.

[23] Satoh K, Kaneko K, Hirota M, Masamune A, Satoh A, Shimosegawa T (2001). Expression of survivin is correlated with cancer cell apoptosis and is involved in the development of human pancreatic duct cell tumors. Cancer.

[24] Kami K, Doi R, Koizumi M, Toyoda E, Mori T, Ito D (2004). Survivin expression is a prognostic marker in pancreatic cancer patients. Surgery.

[25] Ekeblad S, Lejonklou MH, Stålberg P, Skogseid B (2012). Prognostic relevance of survivin in pancreatic endocrine tumors. World J Surg.

[26] Ren YQ, Zhang HY, Su T, Wang XH, Zhang L (2014). Clinical significance of serum survivin in patients with pancreatic ductal adenocarcinoma. Eur Rev Med Pharmacol Sci.

[27] Li Y, Ma X, Wu X, Liu X, Liu L (2014). Prognostic significance of survivin in breast cancer: meta-analysis. Breast J.

